# Note on the Choice of a Sensitivity Weight In Precision Weighing

**DOI:** 10.6028/jres.092.020

**Published:** 1987-06-01

**Authors:** R. S. Davis

**Affiliations:** National Bureau of Standards, Gaithersburg, MD 20899

**Keywords:** mass metrology, precision weighing, sensitivity weight, substitution weighing, transposition weighing, weighing

## Abstract

Good weighing practice usually dictates that, when using double-substitution weighing to determine the mass difference between two weights, the nominal value of the sensitivity weight used to calibrate the optical scale of the mass comparator be at least four times greater than the difference of the two weights being compared. However, there are times when other considerations must override this rule. We examine the theoretical basis for the rule and the penalty for violating it. Finally, we propose a modi-fied weighing scheme which imposes a much less stringent rule for the size of the sensitivity weight. The new scheme requires an additional balance reading, but does not increase the overall measurement time significantly.

## 1. Introduction

Many precision mass comparisons, especially in the realm of metrology, still rely on mechanical balances. These balances may be either one-pan or two-pan. In both cases, however, weighing is done by double substitution between the unknown and an external standard. The procedure in use in most metrology laboratories is shown in table 1.

where *Y* represents the standard, *X* the unknown, and *d* the sensitivity weight. We are assuming that for two-pan balances double substitution has been used rather than double transposition. The arguments that follow apply with modification to the latter technique.

The difference in mass between *Y* and *X*, Δ*M*, (ignoring buoyancy corrections) is sometimes computed as [1]^1^:

$$\Delta M=\frac{I_{1}-I_{2}-I_{3}+I_{4}}{2\left(I_{3}-I_{2}\right)}m_{d}=\frac{\Delta I}{\Delta I_{d}}m_{d}.$$ (1)

We may think of eq (1) as the product of the difference between *Y* and *X* in scale units, (*I*_1_ − *I*_2_ − *I*_3_ *+ I*_4_)/2, multiplied by the balance sensitivity, *m_d_*/(*I*_3_ − *I*_2_). The sensitivity is the proportionality factor which converts differences in scale indication to units of mass. Here *m_d_* is the known mass of *d.*

Most balance indications drift with time. Often the time dependence of the drift can be assumed to be linear. Based on this assumption, one usually tries to make the time intervals between the four weighing operations equal. If this is done, the estimate of Δ*I*, the difference between *Y* and *X* in scale units, will be unbiased by the drift. This will not be true of *I*_3_ *− I*_2_ however. The latter quantity estimates Δ*I_d_*, the value of the sensitivity weight in scale units.

In order to remove the bias, a modified equation is used:

$$\Delta M=\frac{I_{1}-I_{2}-I_{3}+I_{4}}{I_{1}-3I_{2}+3I_{3}-I_{4}}m_{d}.$$ (2)

This is the equation found in the NBS MASS-CODE [2] and has been advocated for general use if the added computational complexity can be handled by computer [3].

## 2. Variance of Δ*M*

There is a general rule [1,3] which states that the metrologist should take care that

$$\frac{\Delta I}{\Delta I_{d}}\;⩽\;0.25.$$ (3)

If the rule is violated, the NBS MASSCODE prints a warning message along with the final calculation [2]. Since the author has not found a rigorous theoretical basis for the rule in the literature, one will now be given.

Each reading of scale indication is subject to random error. Let us assume this error can be characterized by a variance *σ_I_*^2^ which is the same for all measurements. Then the variance of Δ*M* as computed by eq (2) using first order propagation of error techniques is

$$\mathrm{var}\left(\Delta M\right)=S^{2}{\sigma_{I}}^{2}\left[1+5{\left(\frac{\Delta I}{\Delta I_{d}}\right)}^{2}\right]$$ (4)

where *S = 2m_d_*/(*I*_1_ − 3*I*_2_+3*I*_3_ − *I*_4_) is the nominal value of the balance sensitivity (the quantity *m_d_* is treated as a constant in this computation since its variance is usually much smaller than *σ_I_*^2^). Therefore, the rule represented by eq (3) implies that the variance in a single measurement of Δ*M* should not be allowed to increase by more than a factor of 1.31 above its minimum value. The choice of 1.31 is, of course, somewhat arbitrary. Reasonable people might all agree that a factor of 2, for instance, would be intolerably large, while a factor of 11 would be impractically small. We choose 1.31 because it is the *de facto* choice of the NBS MASS-CODE. The important point is that we now have a rational criterion by which to compare various weighing procedures with respect to their demands on the value of the sensitivity weight.

The absence of a term linear in Δ*I*/Δ*I_d_* in eq (4) shows that the estimate of Δ*I* is uncorrelated with the estimate of Δ*I_d_.* It is also evident that the variance of Δ*M* increases monotonically as the ratio Δ*I*/Δ*I_d_* becomes larger. In particular, if Δ*I*/Δ*I_d_* is of the order of 0.5 then the variance of Δ*M* increases to 2.25 times its minimum value. This is unacceptably large in many cases. A value of 0.5 for Δ*I*/Δ*I_d_* was the unavoidable case, however, for a series of important measurements made several years ago on our best kilogram comparator [4]. In order to cope with such a large value of Δ*I*/Δ*I_d_* it was necessary to use a modified weighing scheme.

## 3. The Five Observation Scheme

The weighing scheme used is identical to that of table 1 except for the addition of a fifth operation which is a repeat of the first.^2^ The scheme is shown in table 2.

The apparent difference in mass between *Y* and *X* is then estimated as follows:

ΔM=I1−I2−I3+I4−I2+I3+I4−I5md. (5)

Equation (5) is also unbiased for a linear drift between measurements (though eq (5) is not the least squares solution for a linear drift model). The real virtue of eq (5) is that it is also an unbiased solution for a model which assumes only that the drift between operations 1 and 2 equals the drift between operations 3 and 4; and that the drift between operations 2 and 3 equals the drift between operations 4 and 5 [5]. The first drift occurs between operations which exchange the test weights on the balance pans. The second drift occurs when the sensitivity weight is added or removed. This model frees the operator from having to wait equal times between all measurements. Since the addition or removal of the sensitivity weight is a faster operation than the exchange of test weights, it is usually possible to accomplish the scheme of table 2 (where one need not wait equal intervals between operations) in about the same time as it takes to carry out the scheme of table 1 (where one must take measurements at equally spaced intervals).

When one computes the variance of Δ*M* based on eq (5) one discovers a remarkable result:

$$\mathrm{var}\left(\Delta M\right)=S^{2}{\sigma_{I}}^{2}\left[1-\frac{1}{2}\frac{\Delta I}{\Delta I_{d}}+{\left(\frac{\Delta I}{\Delta I_{d}}\right)}^{2}\right]$$ (6)

where *S* = 2*m_d_*/(−*I*_2_ + *I*_3_ + *I*_4_ – *I*_5_).

The appearance of a term linear in Δ*I*/Δ*I_d_* indicates that, unlike eq (2), the estimate of Δ*I* in eq (5) is not independent of the estimate of Δ*I_d_.* The result of a negative term in eq (6) is that the variance of Δ*M* is insensitive to the ratio Δ*I*/Δ*I_d_* for ratios between 0 and 0.5. Within this range, the variance of Δ*M* is actually below what it would be if the ratio Δ*I*/Δ*I_d_* were zero (table 3). The minimum value for the variance of Δ*M* occurs for the ratio Δ*I*/Δ*I_d_* = 0.25, although this minimum is only 6 percent below the variance for a ratio of zero. Finally, if we want to ensure the variance of ΔM not exceed (1.31) · *S*^2^*σ_I_*^2^, we would make the rule that

$$\frac{\Delta I}{\Delta_{d}}<0.86$$

This should be compared with eq (3).

## 4. Averaging

One of the ways to lessen the dependence of results obtained from table 1 on the ratio Δ*I*/Δ*I_d_* is by averaging. For *N* double substitutions at the same nominal load, one can average the *N* estimates of sensitivity and use the average value in the calculations of the various Δ*M*’s. The NBS MASSCODE takes this approach and amends the rule for the ratio of Δ*I*/Δ*I_d_* to:

$$\frac{1}{N^{1/2}}\frac{\Delta I}{\Delta I_{d}}<0.25$$ (7)

to cover cases where *N* > 1.

The amended algorithm leads to the following variance:

$$\mathrm{var}\left(\Delta M\right)=S^{2}{\sigma_{I}}^{2}\left[1+\frac{5}{N}{\left(\frac{\Delta I}{\Delta I_{d}}\right)}^{2}\right].$$ (8)

There are two possible objections to this approach. First, although the quadratic term in eq (8) is a factor of 1/*N* smaller than the same term in eq (4), it has been converted from a “within” to a “between-time” component [6]. Second, and more serious, the sensitivity of precision mechanical balances may be a function of time. This is certainly the case for NBS-2, the kilogram comparator which was designed and built at NBS and is now in use at the International Bureau of Weights and Measures (BIPM) [7]. In such cases, use of an average value for the sensitivity is unjustified.

## 5. Conclusion

The usual admonition that the ratio Δ*I*/Δ*I_d_* not exceed 0.25 ensures that the variance of a double substitution does not grow by more than 31 percent above its minimum value. We have examined a five-operation weighing scheme and have shown that use of this scheme relaxes the rule to the ratio Δ*I*/Δ*I_d_* not exceeding 0.86. We have also argued that the five-operation scheme can usually be performed in the same amount of time as the more usual four-operation scheme.

As a final comment, we emphasize that this analysis applies to un-servoed mechanical balances. For balances under servo control, the linear range of the scale is usually so large that it is never a problem to meet the conventional ratio rule. In addition, the sensitivities of servo-controlled balances are usually very stable over the course of a series of measurements.

## Figures and Tables

**Table 1:** Four observation scheme.

Operation	Load on Balance	Balance Indication
1	*Y*	*I*_1_
2	*X*	*I*_2_
3	*X+d*	*I*_3_
4	*Y+d*	*I*_4_

**Table 2:** Five observation scheme.

Operation	Load on Balance	Balance Indication
1	*Y*	*I*_1_
2	*X*	*I*_2_
3	*X+d*	*I*_3_
4	*Y+d*	*I*_4_
5	*Y*	*I*_5_

**Table 3:** Comparison of variances with respect to Δ*I*/Δ*I_d_* for results derived from eqs (1, 2, and 5).

Δ*I*/Δ*I_d_*	var(Δ*M*) = k*S*^2^*σ_I_*^2^ k		eq (5)
	eq (1)	eq (2)	
0	1.00	1.00	1.00
1/4	1.12	1.31	0.94
1/3	1.22	1.54	0.94
1/2	1.50	2.25	1.00
1	3.00	6.00	1.50

## Footnotes

1 Figures in brackets indicate literature references.

2 To the author’s knowledge, the first reported use of this weighing scheme was in a 1967 paper by Bowman, Schoonover, and Jones [8]. These authors used a five-observation scheme to compare an external object with the built-in weights of a single-pan, mechanical balance.
